# Decreasing postoperative opioid use while managing pain: A prospective study of men who underwent scrotal surgery

**DOI:** 10.1002/bco2.12

**Published:** 2020-03-20

**Authors:** Aubrey B. Greer, Libert Ramos, Justin M. Dubin, Ranjith Ramasamy

**Affiliations:** ^1^ Department of Urology University of Miami Miller School of Medicine Miami FL USA

**Keywords:** pain, post‐operative, scrotal, surgery, varicocelectomy, vasectomy

## Abstract

**Objective:**

To compare postoperative pain control among men who received different quantities of narcotic prescriptions following scrotal surgery. We hypothesized that men receiving eight vs four pills of acetaminophen 300 mg/codeine 30 mg there would be no significant difference in mean pain following scrotal and inguinal surgery.

**Patients and methods:**

In this prospective, open‐label study, men who underwent scrotal surgery received eight or four acetaminophen 300 mg/codeine 30 mg pills. Men were encouraged to take scheduled non‐steroidal anti‐inflammatory drugs (NSAIDs), apply ice on the incision, and take acetaminophen 300 mg/codeine 30 mg as needed for breakthrough pain. Men were evaluated within 1‐2 weeks after surgery. Statistical analysis was performed using Microsoft Excel and Stata/IC 15.1.

**Results:**

A total of eighty‐seven men met inclusion criteria, fifty‐four men received eight acetaminophen/codeine pills, and thirty‐three men received four pills. There was no significant difference in mean pain score (0‐10) of men receiving eight pills vs four pills in the week after surgery (3.6 ± 1.9 vs 3.3 ± 1.8, *P* = .5004). Of men who used NSAIDs and ice, 93.5% and 92.3% found them to be moderately or very helpful.

**Conclusion:**

Reducing the total prescription of combined narcotic/non‐narcotic medication is not associated with increased postoperative pain in patients undergoing scrotal/inguinal surgery. There was no difference in postoperative pain in men taking eight or four acetaminophen 300 mg/codeine 30 mg pills. A limited prescription of eight or four pills was adequate for pain control in the majority of men who underwent scrotal surgery. NSAIDs and ice were found to be useful adjuncts for pain relief by those who used them.

## 1 INTRODUCTION

The opioid epidemic has been a growing worldwide concern in recent years despite programs and efforts within the healthcare system to better regulate opioid use. Opioid abuse in the United States is the leading cause of injury‐related death, with 1.5 times more overdose deaths than motor vehicle accidents in 2014.^1^ Postoperative pain management has been a difficult area where surgeons aim to provide sufficient pain management without overprescribing opioids. There is currently very little objective data to guide prescribing practices, which makes postoperative management vary amongst practicing physicians.^2^ As a result, surgeons often overprescribe opioids before discharge to avoid the hassle of refilling controlled medications without knowing how much medication their patients truly require.^3^ Studies have found that across surgical specialties, 60%‐80% of men treated with opioids for postoperative pain have left over pills, which they typically keep.^2^, ^4^, ^5^, ^6^ Many patients do not safely store or dispose of the excess medication, which creates a high risk for diversion of narcotics.^7^ Moreover, the duration of opioid prescription rather than the dosage is more strongly associated with misuse.^8^ Despite efforts to curtail excessive opioid prescriptions from a record high in 2010, prescriptions remain three times higher than 1999 levels.^9^

Physicians have a responsibility to treat postoperative pain but there is little objective data to guide prescribing practices. A “one size fits all” approach to postoperative pain control has not worked, so new, more focused postoperative pain guidelines need to be tailored toward the invasiveness, location of the procedure, and expected recovery time. The maximum number of narcotic pills afforded for same‐day surgery by our institutional guidelines is eight pills. Therefore, we started with eight pills of acetaminophen 300 mg/codeine 30 mg and hypothesized that that a 50% reduction in prescribed opiates would offer similar pain relief and that there will be no difference in mean pain scores among men undergoing scrotal and inguinal surgery who receive eight vs four pills.

## 2 PATIENTS AND METHODS

In this institutional review board approved study, informed consent was obtained from study participants. We create a prospective database of men undergoing scrotal and inguinal urological procedures from September 2018 to September 2019 associated with specified current procedural terminology codes: sub‐inguinal varicocelectomy, vasovasostomy, vasoepididymostomy, testes biopsy, microepididymal sperm aspiration, microdissection testicular sperm extraction, scrotal orchiectomy, and hydrocele. In addition to general anesthesia, all men received local anesthesia with 10cc of 1% lidocaine at the end of the procedure. Men were instructed to take non‐steroidal anti‐inflammatory drugs (NSAIDs) every 4‐6 hours, apply ice packs to the incision for 24 hours after surgery, and take 1‐2 pills of acetaminophen 300 mg/codeine 30 mg as needed for breakthrough pain. Initially all men were prescribed eight acetaminophen 300 mg/codeine 30 mg pills, starting at the study's chronological mid‐point (April 2019) the number of pills prescribed was reduced four. This study was open‐label, both participants and clinicians had access to the dose and quantity of prescribed medications.

At the first follow‐up visit, typically within 2 weeks after surgery, the men were asked to recall their mean pain on a scale of 0‐10 with 10 being the worst, the number of narcotic pills taken, whether NSAIDs and ice were used, and the efficacy of the ice and NSAIDs in pain control during the first week post‐surgery. Efficacy was measured using a 4‐point questionnaire as “very helpful,” “moderately helpful,” “minimally helpful,” and “not used”. We attempted to contact men who did not appear at the follow up‐visit via telephone. Men were asked to bring remaining medication to the follow‐up visit for verification or count the remaining pills when contacted by phone. The only exclusion criteria were those who had pre‐existing opioid prescriptions or underwent more than one surgery. A combination of Microsoft Excel and Stata/IC 15.1 was used to organize data and perform statistical analysis. One‐way ANOVAs and independent *t*‐tests were computed to analyze whether the amount of pain or pill consumption varied by age, procedure, and compare levels of pain by number of pills taken. Chi‐squared test were performed to analyze the helpfulness of ice and NSAIDs reported by different groups. Pearson correlations were calculated to examine the degree to which pain and pill consumption correlated. Post‐hoc power calculations were conducted using the freely available ClinCalc post‐hoc power calculator (http://clincalc.com/stats/Power.aspx).

## 3 RESULTS

A total of 127 men underwent scrotal and inguinal surgery. Of those patients, 87 men met inclusion criteria, 2 were excluded due to pre‐existing opioid prescriptions, 2 was excluded due to multiple procedures, and 36 were excluded due to lost to follow‐up. The number of patients lost to follow‐up was similar in both groups. Of the men who met inclusion criteria, 54 men received eight acetaminophen/codeine pills and 33 received four pills. The mean age was 36.8 ± 9.9 years (Table 1). The overall reported mean pain (0‐10) was 3.5 ± 1.9 in the week after surgery, and 3.6 ± 1.9 vs 3.3 ± 1.8 (*P* = .5004) for men receiving eight vs four acetaminophen/codeine pills, respectively (Table 2). Men who received eight pills took a mean of 4.2 ± 3.0 and kept 3.5 ± 3.0 pills (Table 2). There were no phone calls to the provider requesting re‐fills in either group, and none of the men reported receiving additional prescription from outside providers. Men who received four pills took a mean of 2.1 ± 1.8 and kept 2 ± 1.6 pills (Table 2). Forty‐three men (79.6%) who received eight pills and twenty‐eight men who received four pills (84.8%) reported a pain score ≤5/10 in the week after surgery. There was a positive correlation between mean pain and number of pills taken (*r* = 0.6533) for men who received eight pills, meaning that men with higher pain scores generally took more pills. There was no correlation between mean pain and number of pills taken (*r* = 0.1158) for men who received four pills. Overall, 70.1% of men had excess pills (72.7% who received four pills, 68.5% who received eight pills) kept them and did not dispose of them. One‐way ANOVA demonstrated no significant difference in number of pills taken by age (*P* = .1008), or procedure (*P* = .2138). The distribution of unilateral vs bilateral varicocelectomy was equal between the two groups. Among the cohort prescribed four pills, those who took all four pills reported a mean pain of 3.4 ± 1.9 compared to 2.9 ± 2.1 for those who took zero pills. NSAIDs were used by 35.6% of men, of whom 93.5% found NSAIDs to be moderately or very helpful (Table 3). Ice was used by 89.7% of men, of whom 92.3% found ice to be moderately or very helpful. There were similar rates of NSAID and ice usage and efficacy reported between the two cohorts.

**TABLE 1:** Characteristics of men undergoing scrotal and inguinal surgery during the study period

Characteristics	All	8 Pills	4 pills
Age	36.8 ± 9.9	38.0 ± 10.3	34.6 ± 8.8
Procedure			
Varicocelectomy	54	31 (57.4%)	23 (69.7%)
VV/VE	13	11 (20.4%)	2 (6.1%)
Sperm retrieval	16	9 (16.7%)	7 (21.2%)
Hydrocele	3	2 (3.7%)	1 (3%)
Scrotal orchiectomy	1	1 (1.9%)	0 (0%)

**TABLE 2:** The mean pain, number of pills taken and kept in each cohort (8 pills vs 4 pills)

	8 pills	4 pills
Mean pain	3.6 ± 1.9	3.3 ± 1.8
# Pills taken	4.2 ± 3.0	2.1 ± 1.8
# Pills kept	3.5 ± 3.0	2 ± 1.6

**TABLE 3:** The utility of ice and NSAIDs in reducing pain in the week after scrotal and inguinal surgery

	Total		8 Pills		4 Pills	
	Ice	NSAIDs	Ice	NSAIDs	Ice	NSAIDs
Not used	10.3% (9)	64.4% (56)	13.0% (7)	63.0% (34)	6.1% (2)	66.7% (22)
Minimally helpful	6.9% (6)	2.3% (2)	7.4% (4)	1.9% (1)	6.1% (2)	3.0% (1)
Moderately helpful	13.8% (12)	8.0% (7)	20.4% (11)	5.6% (3)	3.0% (1)	12.1% (4)
Very helpful	69.0% (60)	25.3 (22)	59.3% (32)	29.6% (16)	84.8% (28)	18.2% (6)

## 4 DISCUSSION

Achieving adequate pain control while avoiding overprescribing opioids is a challege for physicians. We conducted a single‐institution prospective study on consecutive men undergoing scrotal and inguinal surgery to evaluate the efficacy of NSAIDs, ice, and a comparison of four vs eight acetaminophen 300 mg/codeine 30 mg tablets for postoperative pain control. Those receiving eight vs four pills reported similar mean pain (3.6 ± 1.9 vs 3.3 ± 1.8) and there was no statistically significant difference (*P* = .5003) (Table 2).

Despite the limited prescription, 46.6% of opioid pills from filled prescriptions went unused and 70.1% of men who filled the prescription had excess acetaminophen/codeine remaining. Interestingly, these rates are similar for both cohorts. Nearly half the pills prescribed to the eight‐ and four‐pill cohorts were excess (45.9% vs 48.5%) and most were kept for later (72.7% vs 86.5%). This is consistent with other recent studies on opioid prescriptions after major urological surgeries which found that 60%‐80% of prescription opioids went unused and were kept by the patients.^2^, ^6^, ^10^

Ice and NSAIDs are useful adjuncts for pain control for most men, 89.7% and 93.5% of those who used them found ice and NSAIDs to be moderately to very helpful. The rates of ice and NSAID use and reported efficacy were similar between the two cohorts (Table 3). Interestingly, four‐pill cohort reported greater satisfaction with ice than the eight‐pill cohort (84.8% found ice to be very helpful vs 59.3% in the eight‐pill cohort).

Recent studies have demonstrated success in opioid‐free postoperative pain control. A retrospective review of patients undergoing cystoscopy found that prescribing patients diclofenac instead of hydrocodone or oxycodone resulted in nearly identical number of calls for postoperative pain issues.^11^ Non‐narcotic management of postoperative pain following pediatric robotic pyeloplasty showed shorter length of stay without increase in complications.^12^ A study of postoperative pain among men and women who underwent surgery found that those who received only ketorolac in the postanesthesia care unit generally experienced less pain than those receiving metamizole, morphine, or tramadol.^13^

In light of these studies and our own results, we believe a reasonable next step would be to attempt narcotic‐free postoperative management of men who have undergone scrotal and inguinal surgery. There was no statistically significant difference in pain reported by those receiving eight vs four acetaminophen/codeine pills, and both groups took approximately half the pills prescribed on mean (4.2 ± 3.0 vs 2.1. ± 1.8). In our study, many more men took acetaminophen/codeine (78.1%) than NSAIDs (35.6%), despite the high efficacy reported by those who took NSAIDs (93.5%). A possible explanation is that some patients who took all the opioid pills believed that they were following physician orders to take them all. Another possible explanation is that anticipation of pain prompted men to take the medication perceived to be stronger. The low overall use of NSAIDs (37.0%) vs acetaminophen/codeine (79.6%) suggests possible confusion with physician instructions or patient's desire for a stronger medication, or both. Furthermore, 16 of 25 men who took all prescribed acetaminophen/codeine pills reported a mean pain of ≤4, which may reflect the pills efficacy or once again, a misunderstanding of instructions. Our data suggests that the men did not take scheduled NSAIDs with acetaminophen/codeine for breakthrough pain as instructed, and that NSAIDs alone may be sufficient if properly utilized. The importance of clear postoperative instructions is one of the lessons we learned and is imperative successful pain control.

Limitations of our study include dropout and lost to follow‐up, patient recall bias, heterogeneity of procedures, sample size, and single‐institution, single surgeon. The number of patients lost to follow‐up was similar in both groups. Despite these limitations, this is a novel study that successfully assessed the adequacy of postoperative pain management with opioids in men who underwent scrotal and inguinal surgery.

## 5 CONCLUSION

Reducing the total prescription of combined narcotic/non‐narcotic medication is not associated with increased pain in patients undergoing scrotal/inguinal surgery. There was no difference in postoperative pain in men taking eight or four acetaminophen 300 mg/codeine 30 mg pills after scrotal and inguinal surgery. NSAIDs and ice were found to be useful adjuncts for pain relief by those who used them. NSAIDs in particular were found to be helpful by nearly all men but underutilized. Underutilization of NSAIDs may be due to patient misunderstandings of postoperative pain management instructions, and therefore it is important for physicians to make sure directions for medication usage are clear prior to discharge in order to reduce opioid usage.

## CONFLICT OF INTEREST

The authors have no potential conflicts of interest to disclose.

## 6 ETHICS STATEMENT

This present study protocol was reviewed and approved by the institutional review board of the University of Miami (ID: 20180809). Informed consent was submitted by all subjects.

## AUTHOR CONTRIBUTIONS

Conceptualization: Ranjith Ramasamy. Data curation: Aubrey Greer, Libert Ramos. Formal Analysis: Aubrey Greer. Funding acquisition: NA. Investigation: Aubrey Greer, Libert Ramos, Ranjith Ramasamy. Methodology: Aubrey Greer, Libert Ramos, Ranjith Ramasamy. Software: Stata/IC 15.1, MS Excel, MS Word. Supervision: Ranjith Ramasamy. Writing‐original draft: Aubrey Greer, Justin Dubin. Writing‐review and editing: Ranjith Ramasamy, Justin Dubin, Aubrey Greer.
